# Supplementary material for: Living through Cyclone Freddy: “Gendered Health Impacts and Coping Responses in Malawi”
Source: PLOS Glob Public Health. 2026 May 7;6(5):e0005799. doi: 10.1371/journal.pgph.0005799 (PMC13152185; doi:10.1371/journal.pgph.0005799)
Supplement: Attachment — Submitted filename: PGPH-D-25-03980-AAA Reviewer.pdf [file pgph.0005799.s002.pdf]

PLOS Global Public Health

Living Through Cyclone Freddy: “Gendered Health Impacts and Coping Responses in Malawi”

--Manuscript Draft--

Manuscript Number:	PGPH-D-25-03980
Article Type:	Research Article
Full Title:	Living Through Cyclone Freddy: “Gendered Health Impacts and Coping Responses in Malawi”
Short Title:	Gendered Health Impacts of Cyclone Freddy in Malawi
Corresponding Author:	Chipi Payesa<br>Kamuzu University of Health Sciences: University of Malawi College of Medicine<br>Blantyre, Blantyre MALAWI
Order of Authors:	Chipiliro Payesa
	Edith Milanzi
	Junious Sichali
	Ruth Holla
	Thelma Kaliu
Keywords:	Climate Change, Gender, Public Health, Disaster
Abstract:	Malawi has increasingly faced severe climate-related disasters, with Cyclone Freddy in 2023 among the most devastating in the country’s history. While disasters are often considered gender-neutral events, emerging evidence suggests that women and girls are disproportionately affected across health, social, and economic domains. This study examined the gendered health impacts and the responsiveness of humanitarian interventions during and after Cyclone Freddy in Malawi. We conducted a qualitative study in Blantyre (urban) and Chiradzulu (rural) districts between August and September 2024. Forty-six in-depth interviews were held with survivors, complemented by eight key informant interviews with representatives of governmental and non-governmental organizations involved in the response. Thematic analysis was applied to explore gender-specific experiences, health challenges, and perceptions of response efforts. Findings revealed significant gendered disparities. Women reported heightened loss of reproductive health services, and severe disruption to menstrual hygiene management and exposure to gender-based violence. Maternal health needs were largely unmet, and mental health services were delayed and under-resourced, exacerbating psychological distress among women and girls. Men primarily emphasized loss of livelihoods and inability to provide for families, which contributed to mental health strain. While some humanitarian actors adopted gender-responsive measures, such as dignity kits and separate sanitation facilities, these efforts were inconsistent, often reactive, and limited by funding and planning gaps. Cyclone Freddy underscored the urgent need for gender-sensitive disaster preparedness and response frameworks in Malawi. Integrating gender considerations into health, protection, and recovery strategies is essential to mitigate inequitable impacts on women and girls in future crises.
Additional Information:	
Question	Response
**Financial Disclosure**<br><br>Enter a financial disclosure statement that describes the sources of funding for the work included in this submission and the	Funding for this study was provided by the Gender and Public Health Emergencies The funders had no role in study design, data collection and analysis, decision to publish, or preparation of the manuscript.

role the funder(s) played. This includes grants and any commercial funding of the work or authors.<br><br>This statement will be typeset if the manuscript is accepted for publication.<br><br>*Please review the submission guidelines and the instructions link below for detailed requirements and guidance.*	
**Competing Interests**<br><br>On behalf of all authors, disclose any competing interests that could be perceived to bias this work.<br><br>This statement will be typeset if the manuscript is accepted for publication.<br><br>*Please review the instructions link below and PLOS Global Public Health's competing interests policy to determine what information must be disclosed at submission.*	None to declared
**Data Availability**<br><br>Before publication, Authors are required to make fully available and without restriction all data underlying their findings. Please see our PLOS Data Policy page for detailed information on this policy.<br><br>A **Data Availability Statement**, detailing where the data can be accessed, is required at first submission. Insert your Data Availability Statement in the box below.<br><br>Please see the data reporting section of our submission guidelines for instructions on what you need to include in your Data	Data is available upon request

Availability Statement.<br><br>This statement will be typeset if the manuscript is accepted for publication.<br><br>*PLOS allows rare exemptions to address legal and ethical concerns. If you have legal or ethical restrictions, please use the box below to detail these in full sentences for the Journal team to consider.*	

Manuscript Click here to access/download;Manuscript;Manuscript Final.docx

Living Through Cyclone Freddy: "Gendered Health Impacts and Coping Responses in Malawi"

Short title: Gendered Health Impacts of Cyclone Freddy in Malawi

Authors: Chipiliro Payesa[1] Edith B. Milanzi[1], Junious Sichali[2], Ruth Holla[1,3], Thelma Kaliu[4].

1. Femanalytica. P.O. Box 30815, Lilongwe
2. University of Bath, UK
3. WHO. P.O. Box 30390, Lilongwe 3
4. GenHub Malawi, P. O Box 410 Lilongwe

Keywords: Climate Change, Gender, Public Health, Disaster

Abstract:

Malawi has increasingly faced severe climate-related disasters, with Cyclone Freddy in 2023 among the most devastating in the country's history. While disasters are often considered gender-neutral events, emerging evidence suggests that women and girls are disproportionately affected across health, social, and economic domains. This study examined the gendered health impacts and the responsiveness of humanitarian interventions during and after Cyclone Freddy in Malawi. We conducted a qualitative study in Blantyre (urban) and Chiradzulu (rural) districts between August and September 2024. Forty-six in-depth interviews were held with survivors, complemented by eight key informant interviews with representatives of governmental and non-governmental organizations involved in the response. Thematic analysis was applied to explore gender-specific experiences, health challenges, and perceptions of response efforts. Findings revealed significant gendered disparities. Women reported heightened loss of reproductive health services, and severe disruption to menstrual hygiene management and exposure to gender-based violence. Maternal health needs were largely unmet, and mental health services were delayed and under-resourced, exacerbating psychological distress among women and girls. Men primarily emphasized loss of livelihoods and inability to provide for families, which contributed to mental health strain. While some humanitarian actors adopted gender-responsive measures, such as dignity kits and separate sanitation facilities, these efforts were inconsistent, often reactive, and limited by funding and planning gaps. Cyclone Freddy underscored the urgent need for gender-sensitive disaster preparedness and response frameworks in Malawi. Integrating gender

considerations into health, protection, and recovery strategies is essential to mitigate inequitable impacts on women and girls in future crises.

Introduction

In March 2023, Malawi experienced one of the most intense and deadly tropical cyclones recorded in the Southern Hemisphere Cyclone Freddy. Cyclone Freddy triggered a state of disaster across 14 districts, displacing over half a million people and resulting in more than 1,000 confirmed deaths, with over 500 people reported missing(1) . Its impact followed closely on the heels of Cyclone Ana in 2022 and Cyclone Idai in 2019, compounding the country's vulnerability to repeated climate-related disasters. These events caused widespread destruction to public infrastructure and disrupted access to essential health services, exacerbating public health risks across affected regions(2)

Climate-related disasters in Malawi have increasingly exposed the deep social and health inequities within emergency response systems. While the destruction from such events is extensive, the human toll is far from uniform. Women and girls are disproportionately affected. They face vulnerabilities ranging from increased exposure to gender-based violence, limited access to sanitation and menstrual hygiene, and constrained reproductive health services, to greater burdens of psychological distress and malnutrition during displacement(3).

During Cyclone Freddy, of the more than 2.5 million people affected, 52% were women, including over 100,000 pregnant women, while 65% of the displaced population were

female and 55% were children(4) In previous disasters, such as Cyclone Idai, women also accounted for the majority of those displaced and disproportionately represented household heads among the most affected populations (5)Research from other global contexts also follow this pattern. Following the 2004 Indian Ocean tsunami, women experienced significantly higher mortality rates than men, not because of biological differences, but due to their disadvantaged social positioning during disaster response(3) Similarly, limited privacy in shelters, poor sanitation, and weak integration of women's health needs into emergency responses contribute to compounding vulnerabilities—particularly in settings where access to healthcare is already limited (6,7).

The mental health implications of such disasters also remain poorly documented and often neglected in response frameworks. Traumatic experiences, including the destruction of homes, separation from family, and loss of life, heighten risks for depression, anxiety, PTSD, and other psychological conditions. These effects are especially pronounced among women, who are often exposed to additional stressors such as domestic violence and caregiving responsibilities in the wake of such crises (8).

Despite the recognition of these gendered risks, disaster responses continue to be shaped largely by blanket decision-making processes, with limited incorporation of gender-sensitive needs. Effective disaster health responses must therefore go beyond general preparedness to include tailored provisions for different gender groups(9).

The study, using a qualitative approach, aimed to investigate the gender-specific health impacts of Cyclone Freddy as experienced by survivors in Chiradzulu and Blantyre districts in Malawi. In addition, we also examined the gender-responsive health

interventions implemented by government and humanitarian organizations in response to the crisis and assessed their effectiveness in addressing the differentiated needs of affected populations with the goal to identify best practices that could inform more equitable and gender-sensitive disaster health response strategies in similar low-resource settings.

Methods

Study design and setting

We conducted a qualitative study to examine differentiated health related gender impacts of Cyclone Freddy in Malawi as well as gender responsiveness of the response to the disaster. The study was implemented in two Malawian districts Blantyre and Chiradzulu. These locations were strategically selected to represent both urban (Blantyre) and rural (Chiradzulu) settings, offering a comprehensive view of the disaster's impacts across different geographical and socio-economic contexts. We conducted in-depth interviews with cyclone survivors from various demographic groups, such as men, women, child-headed households, pregnant women, and adolescents, and key informants from humanitarian organizations that led recovery efforts.

Sampling

A purposive sampling technique was used to select participants who had directly experienced the cyclone's impact or had been involved in the response efforts. This

method ensures the inclusion of individuals who provided in-depth and relevant information about the gender-specific challenges and interventions post-disaster. The sampling was also aimed to achieve a balanced representation of the target population, taking into consideration gender, age, and socio-economic status, to gather a wide range of perspectives.

Sample size

Given the qualitative nature of this study, we determined sample size based on the principle of data saturation. This approach entails continuing data collection until no new information is obtained, and redundancy is achieved in the data. In this regard, theoretical saturation was attained after conducting 22 in-depth interviews from Chiradzulu district and 24 from Blantyre making a total of 46, along with 8 key informant interviews with stakeholders.

Data collection

Study data were collected from 22 August 2024 through 22 September 2024 in designated survivor camps. Data was collected through a combination of semi-structured interviews, and key informant interviews. Prior to the main data collection phase, a pilot survey was conducted to assess the clarity, relevance, and appropriateness of the interview guide . Based on feedback obtained during the pilot, modifications were made to improve the effectiveness of the interview guide and ensure participant comfort and comprehension

In-depth interviews with survivors were guided by a thematic framework comprising key domains such as individual experiences during the cyclone, access to healthcare services during and after the disaster, factors influencing healthcare accessibility, and perceived disparities by gender, socio-economic status, and other individual characteristics

Key informant interviews targeted representatives from governmental and non-governmental organizations engaged in the Cyclone Freddy response. These interviews focused on examining the extent to which emergency health interventions addressed gender-specific needs.

Ethics Statement

Approval for this study was provided by the National Committee on Research in the Social Sciences and Humanities under the Malawi National Commission for Science and Technology PROTOCOL NO. P.04/24/868) The participants received written information about the study, their informed consent was sought, and participation was voluntary.

Data analysis

Audio-recorded interviews were transcribed verbatim and translated from Chichewa to English by experienced researchers. Analysis was conducted using an inductive thematic analytical approach with NVivo 14(10) This approach enabled the identification, analysis, and reporting of response patterns, aiding in the organization, description, and detailed interpretation of the information. It was an iterative process in which researchers immersed

themselves in the data by reading transcripts multiple times to develop a codebook for data coding. The research team then created themes and sub-themes, which were interpreted and labelled accordingly. To ensure the validity of the findings, the research team conducted repeated reviews of the process to guarantee that the findings were credible, consistent, neutral, and applicable.

Findings

Participant characteristics

Of the 46 participants, 22 (47.8%) were from Blantyre and 24 (52.2%) were from Chiradzulu districts. The mean age (SD) of the participants was 46.2 (14.6) years in Blantyre and 35.6 (12.9) year. majority were married when the cyclone occurred, and most participants had either attended primary or secondary school education.

Table 1. Characteristics of participants by district*

**Characteristics**	**Blantyre (N=22)**	**Chiradzulu (N=24)**	**Total (N=46)**
Age (mean, SD)	46.2 (14.6)	35.6 (12.9)	40.7 (14.7)
Sex, female, (n, %)	12 (54%)	11 (45%)	23 (50%)
Marital Status, (n, %)			
Married	10 (45.5%)	19 (79.2%)	29 (63.1%)
Widowed	5 (22.7%)	2 (8.3%)	7 (15.2%)
Divorced	3 (13.6%)	0 (0)	3 (6.5%)
Single	4 (18.2%)	3 (12.5%)	7 (15.2%)
Highest Level of Education (n, %)	1 (4.6%)	0 (0)	1 (2.2%)
None	4 (18.2%)	17 (70.8%)	21 (45.6%)
Primary school	11 (50.0%)	7 (29.1%)	18 (39.1%)

Secondary school	6 (27.2%)	0 (0)	6 (13.0%)
Tertiary			

*Percentages calculated out of column totals.

A thematic analysis of the data generated six major themes: Impact of Cyclone Freddy, Health and Well-being in the camps, Gender and Vulnerability Issues, Emergency Response and Support, Community and Partner Collaborations, as well as Preparedness and Future Resilience. These main themes are described below.

Results

1. Immediate Physical Health and Displacement

The immediate period following Cyclone Freddy revealed pronounced gendered disparities in displacement experiences. Women emphasized the dual burden of physical danger and familial separation. One female participant reflected,

"It was very painful for me, and it also felt like my marriage was broken because my husband and I were in different camps" (Female, 29, Chiradzulu).

This sense of relational rupture compounded women's emotional distress during evacuation. Beyond separation, women often bore the responsibility of caring for injured relatives even as they struggled to find safe shelter as narrated by one participant.

"My mother was badly injured in the ribs; she was cut by iron sheets in the ribs, armpits and the body looked like she was beaten. She says she could see the Chanza River close, but she was stuck in the small river and that's what saved her. Also, she was hit by house bricks and rocks as the water was sweeping her away. She was badly injured and to see her now alive, we call it all mercy."
(Female 27, Chiradzulu)

Men's accounts, while acknowledging similar physical injuries prioritized material loss and livelihood risks. A male respondent described,
"*Houses, I had three houses. All were lost. My house and another semi-detached they were all swept by the water. Everything was lost!... We are just grateful to GOD that we are alive*" (Male, 64, Blantyre).

Similarly, as described by a 25-year-old participant
"All my belongings which were in the house were swept away by the water and mud, things like cooking utensils, beddings, and, as I have said earlier, that I do business. My sausage-making machine was carried away by water. What I am very worried about is the machine because my life now is difficult compared to how things were before. I don't think I will ever find the machine again." (Male 25, Blantyre)

Such narratives expose how asset destruction exacerbated men's psychosocial trauma, as the loss of homes and tools felt like a loss of personal and economic identity.

Logistical challenges in camp registration further unequally impacted women, who often assumed primary caregiving roles. In Chiradzulu, a woman described her assuming caring roles for her sister's:

"The cyclone affected me because I used to stay with my sister but unfortunately, her leg got cut off during the cyclone while she was running away. She doesn't walk properly now because she lacks a wheelchair. So, she drags herself when walking. … She was examined, and they recommended an artificial leg, but it hasn't come up to date. We asked for a wheelchair, but no wheelchair came. So, she had difficulties in walking. She cannot even indulge in business because she cannot cover much ground by dragging herself." (Female, 36, Blantyre)

2. Access to Maternal Health Services

Access to health services in camps following Cyclone Freddy was inconsistent, with gender-specific health needs often overlooked in the early stages. While some essential services such as malaria treatment, blood pressure checks, and family planning were intermittently available, coverage varied widely.

"*There was a hospital… you were able to receive family planning services… malaria medication if diagnosed," shared a woman in Blantyre* (Female, 52).

However, facilities were unevenly staffed and resourced across camps.

Government-deployed mobile health teams which provided structured visits in some locations, enhancing service delivery and building trust. As one man explained,

“*Health workers were coming every morning and afternoon… asking about your problem, you were helped accordingly*” (Male, 45, Chiradzulu).

Despite these efforts, many survivors avoided formal clinics due to fears of infection or overwhelmed systems, opting instead for over-the-counter remedies or informal advice. Chronic illnesses such as asthma and HIV received little attention. Patients were referred to distant health centres lacking transport support.

“I’m asthmatic… they had no asthma medication. I still had to travel to the health centre,” noted one woman (Female, 45, Blantyre).

For women living with HIV or using injectable contraceptives, the disruption in care threatened continuity and health outcomes.

Adolescent women, pregnant and postpartum women were especially underserved. Maternal services were not prioritized, and pregnant women queued alongside others without special consideration. Two participants below highlight this concern

Some services are special for women, and some are specific to girls. For example, services like family planning and good maternal well-being. It wouldn’t be good for a pregnant woman to share a clinic with men. Also, nowadays, many girls start using contraceptives as a way of protecting themselves. So, they wouldn’t be comfortable getting contraceptives with men around. They would be shy. Hence, they would rather not get the contraceptives than to be seen by men. Consequently, they indulge in sex without contraceptives, thereby risking their lives.”

(Female 42, Chiradzulu)

“Regarding my family's well-being, I can say that my wife miscarried after the accident. At that time, it was hard for women to access various services or aid. Like sanitary pads, for instance, it was hard for women to get, and some were on injectable contraceptives. So, during the time of the accident, their focus was on healing from all this and forgetting about contraceptives because their heads were saturated.”

(Male 25, Chiradzulu)

Stakeholders acknowledged that their health responses lacked a gender-responsive framework. While some later adjustments, such as the deployment of female health workers, improved access to reproductive care, these were reactive rather than planned. As one key informant concluded,

“We responded, but not from a gendered understanding of health needs.”- Key informant, Humanitarian

Reproductive health needs were severely disrupted in the aftermath of Cyclone Freddy, with displaced women facing numerous challenges in accessing family planning services, antenatal care, and safe spaces for delivery. These disruptions were compounded by increased risks of gender-based violence (GBV), particularly in overcrowded camps where law enforcement and protection systems were stretched thin.

Key informants confirmed these patterns. *“*

We observed that SRH services collapsed entirely in the first weeks. Pregnant women had no follow-up, and those on family planning dropped off. In such conditions, even

those using long-term contraceptives became anxious without access to health professionals" (KII 5).

WASH (Water, Sanitation, and Hygiene) and Menstrual Hygiene Challenges

Access to safe and gender-sensitive WASH facilities was severely compromised in the aftermath of Cyclone Freddy. Overcrowded camps, damaged infrastructure, and limited resources created widespread hygiene risks, particularly for women and girls. Shared sanitation facilities lacked privacy and cleanliness, often forcing women to use the same toilets as men, which compromised both dignity and health.

"*There wasn't any cleanliness in the facilities, it was very unhygienic…We didn't have enough clean water either as the geographic condition of this area makes it difficult for us to have access to clean piped water from water board due to low water pressure. Most of the time we only receive piped water during the night, so people use wells and boreholes as alternate source of water. Water from these alternative sources is mostly unhygienic and normally go bad in a few days if kept in buckets."*

(Cyclone Freddy Survivor Male 43, Blantyre)

Overcrowded camps and damaged infrastructure led to shared and unsanitary toilet spaces, limiting privacy for women and girls. Women often had to use the same toilets as men, even during menstruation, heightening discomfort and hygiene concerns.

"*There were toilets for both males and females… some women would still go to the male toilets while in her period. So, they would stain and thereby compromise hygiene*"

(Cyclone Freddy survivor Female, 42, Chiradzulu).

Water scarcity worsened the situation. Unreliable piped water forced reliance on boreholes and open wells, often of poor quality. Basic hygiene tasks became difficult,

especially for pregnant women and caregivers. In some areas, toilet facilities were blocked and unusable, contributing to disease risks.

"*The sleeping rooms were okay, but unlike the toilets, they were not hygienic… some could defecate on the sides, and it became difficult for someone to utilize them*" (Cyclone Freddy survivor Male, 64, Blantyre).

Menstrual hygiene was especially neglected in the early response. Many women lacked sanitary pads, underwear, and private areas to wash and dry cloth substitutes.

"*After the tragedy… I started menstruation while only dressed in a wrapper… I was given sanitary pads, but it was a few days after*" (Cyclone Freddy survivor Female, 25, Chiradzulu).

Improvisation was common, but it came with social stigma and fears. One woman noted,

"*We would use a piece of cloth… but we would find challenges in where to hang the cloth… some would use those things for ill purposes*" (Female, 20, Chiradzulu).

The lack of private bathing and drying spaces left women feeling exposed and anxious.

Camp layout further restricted dignity. Women were hesitant to clean menstrual cloths openly due to the constant presence of men.

"*It was difficult for us to wash and dry our underwear during periods because men were everywhere*" (Female, 29, Chiradzulu).

While some stakeholders later responded by constructing dedicated women-only toilet blocks, these improvements came late.

"*There were some toilets that were specifically built for women because we understood that… women and girls were the ones who were most affected*" (KII 3). Another key informant expressed how gender needs were accounted for as that is the mandate of their organization "*we do take a gender responsive approach which really aims to address the specific needs and challenges which are faced by the different genders…we provided the menstrual hygiene kits, maternal health supplies which some of the women said they were not enough. (KII 4)"*

While other organizations proactively built separate latrines for women and girls in some camps, female hygiene needs were not necessarily prioritized, and the lack of early planning for menstrual management reflected broader gaps in recognizing women's unique vulnerabilities. Some camps later distributed hygiene kits, but menstrual products were not always included or sufficient. As a key informant explained,

"*Menstruation became a source of shame and distress… they had no privacy and feared their sanitary cloths would be stolen for ill purposes*" (KII 1).

3. Gender-Based Violence

Sexual exploitation emerged as a major concern across the two camps. Transactional sex, driven by food insecurity and economic desperation, was frequently reported. Adolescent girls and young women were particularly vulnerable. Key informants corroborated these

accounts, highlighting that women were not sex workers but were engaging in survival strategies due to a lack of alternative support mechanisms.

“*In these camps, there's a lot of physical and sexual violence because displacement and loss of shelter as well as breakdown in law and order normally leave women exposed and vulnerable. So, the psychological trauma of such experiences can have long-lasting impacts on women's physical and mental well-being (KII 5)*”

As another informant reported

“*This time around we have got babies born from such kind of arrangement.... Which means, even if you ask, how did you get these babies, they will simply say “ah, I was just frustrated... maybe the one that I was sleeping with, was giving me food, was giving me some financial assistance… so, we ended up having unprotected sex, and we ended up getting pregnant (KII 1)*”

This exploitation exposed major gaps in the humanitarian response, particularly in the protection of displaced women.

Predominantly, our findings indicated that organizations, like UNICEF, quickly responded by establishing safe spaces and support mechanisms within the camps. They conducted community education and gender sensitization to empower victims to speak out whenever they encounter violence. However, few incidences of rape and abuse of power were reported in some camps.

“*Reports of gender-based violence were there; it was mostly happening to girls. Like last time, somebody came to visit his relative, he found a young girl aged fifteen and enticed*

her by asking her to accompany the man to buy her some food. The girl went with the man, and she was raped. When she came back, she did not disclose it to people at the camp until some days had passed."

(Female 45, Blantyre)

"*There was rape abuse in the camp. For the chefs especially men at the kitchen, they would entice women to sleep with them in exchange with good food. So, they would tell girls in the camp to have sex with them if they wanted good food like eggs*."

(Male 64, Blantyre)

"*Yes, some people would come and entice young girls/women to sleep with them in exchange for money. Some of these girls would accept because they needed the money, and this became a habit."*

(Male 43, Blantyre)

These accounts highlight the urgent need for integrated, gender-sensitive response mechanisms in disaster settings. Key informants emphasized that GBV services must be embedded in emergency response strategies from the outset, not as supplemental considerations, but as essential, lifesaving services.

However, others described camps where security was wholly inadequate.

"*There was no security, people would come and cause disturbances. At a certain point thieves came and caused panic by threatening the people that another cyclone is coming for the camp. When people tried to escape, the thieves went into the camp and stole their possessions,*" (Male 43, Blantyre).

These contrasting accounts show how inconsistent protection systems resulted in vastly different safety outcomes for displaced populations.

Long-Term Impacts: Psychological, Physical and Mental Health Consequences.

The prolonged aftermath of Cyclone Freddy has deeply affected survivors' mental health, physical well-being, and socio-economic stability. While all survivors endured significant losses, the long-term consequences have manifested in distinctly gendered ways, shaped by social roles, caregiving responsibilities, and access to resources. While most participants acknowledged psychosocial support, most reflected that mental health support was delayed and under-resourced. In the absence of formal psychosocial services, teachers and camp leaders informally identified distressed individuals for referral. However, structured mental health systems were largely absent during critical early periods.

"*Support came, but it came late, Mental health support was supposed to be offered earlier because people were traumatized … there were no structures for the counsellors*," (Male, 57, Blantyre).

For many women, the cyclone fractured not just homes, but a sense of independence. Women reported long-term physical effects from injuries sustained during the cyclone, which have constrained their ability to work. One respondent shared,

"*Before the accident happened, we were physically fit… but now, after the injuries, we were instructed by the hospital to refrain from indulging in heavy manual tasks for three or four years*" (Female, 20, Blantyre).

These physical limitations were often compounded by ongoing caregiving responsibilities, creating a cycle of economic vulnerability.

Mental health challenges extended beyond the individual to entire families. Many women recounted that their children still experience psychological distress during rainy seasons, associating it with the trauma of the cyclone.

"*My last-born still lives in fear during rainy seasons… she got stuck to a wall and survived, but she still lives in fear… the whole family is still psychologically unwell*," said a mother in Chiradzulu (Female, 38, Chiradzulu).

Another participant described how her firstborn son, who watched the cyclone from a tree, later developed hallucinations:

"*He was hallucinating a lot… we had to send him to stay with relatives so he could forget what happened*" (Female, 29, Chiradzulu).

Male participants also articulated long-term psychological impacts,

"Sometimes we think about how we used to live with our parents compared to our living conditions right now. This affects me psychologically to the extent that I have sleepless nights. I sometimes start thinking about these while still in class, which affects my performance" (Male 15, Chiradzulu)

but their narratives were often intertwined with a sense of failed provision. A male participant recounted how his family's business was destroyed:

"*To say the truth, we still have thoughts. Since that incident, a lot of things have become difficult in our lives. Our lives have been affected because the people we used to do business with relocated; things have become expensive, and we don't have money…. if only we had been helped earlier for some months, as they said, then we could have found our feet and become self-reliant.*"
(Male 43, Blantyre)

This may reveal that men's distress frequently stemmed from their inability to fulfil expected roles as economic providers.

Other long-term consequences involved disruptions in chronic disease management. Women, particularly those heading households, were often forced to deprioritize their own or their children's medication due to food insecurity. As highlighted by one key informant *"Female-headed families… could tell you that they have stopped their child from taking medication because they don't have food… they have stayed for 4 days without proper meals*" (KII 1). The loss of privacy and access to health infrastructure further exacerbated these risks, with one woman explaining that many were "*too shy to come up clearly that they lost their medication*" (Female, 45, Blantyre), leading to worsening health outcomes.

While some survivors found ways to cope, such as the woman who trained as a mental health counsellor with Saint John's and now supports others, these cases were the exception rather than the norm. Overall, the evidence paints a complex and unequal

recovery trajectory in which women, especially those with caregiving duties, faced a multi-layered burden: rebuilding their lives while managing unresolved trauma, disrupted health services, and increased social vulnerability.

Key informants consistently underscored that women and girls are likely to experience more profound and long-lasting consequences from Cyclone Freddy due to pre-existing vulnerabilities that were worsened by displacement and loss. Several interviewees emphasized the mental health toll, particularly for women who experienced the deaths of close family members, the collapse of their homes, or exposure to gender-based violence.

Critically, multiple informants stressed that unless mental health and gender-based services are made integral to recovery efforts, women and girls will continue to bear disproportionate burdens, emotionally, physically, and socially.

Gender-Responsiveness of Humanitarian Response

Key informant interviews revealed positive levels of gender responsiveness in the humanitarian response to Cyclone Freddy. Several stakeholders reflected deliberately on the importance of integrating gender considerations into their programming, with some reporting intentional efforts to ensure women and girls were not overlooked in the distribution and design of services.

One stakeholder explained that during needs assessments, their team specifically asked women about the kinds of support they needed, resulting in the inclusion of menstrual hygiene products and reproductive health services:

"*Being an organization that focuses on youth, women and children, we do take a gender responsive approach which really aims to address the specific needs and challenges which are faced by the different genders. When we conducted our assessments, we ensured we included women and girls separately… so that they could voice out what they needed. That's how we knew about the need for dignity kits, or that they were struggling with their menstrual hygiene. We acted on that…we also established safe spaces and support mechanism within the camps, to prevent and respond to GBV*" (KII 5)

This commitment extended to involvement of other partners. Another key informant respondent detailed how they worked with government officials to facilitate gender responsiveness.

"We liaised with the District Disaster Risk Management Committee where they have a designated gender officer, who is responsible to oversee all the gender related issues…we coordinated with this officer to inform our response" (KII 2)

However, one informant admitted that gender was initially not fully integrated into their responses, which limited the effectiveness of some interventions:

"*Honestly, I think our early response was more general though after the cyclone, awareness campaigns were being done, both in the communities and in camps about gender-based violence*." (KII 3)

Discussion

This study offers a grounded understanding of the gendered health impacts of Cyclone Freddy on affected populations in southern Malawi, informed by both survivor accounts and from frontline humanitarian actors. The findings reveal the complex ways in which disaster exposure and recovery are shaped by gender, highlighting both unique vulnerabilities and differences in perceived impact and response between women and men.

Study participants reported several immediate effects of Cyclone Freddy, including displacement, injury, deaths, property damage and disrupted access to essential services. These findings are in line with previous studies(11)]. Similarly, Braka et al, also reporting on the effects of the 2023 Cyclone Freddy, stated that a total of 659,278 individuals were displaced in the affected districts, with the majority of them seeking refuge in schools, temporary shelters, health facilities, churches and mosques(9) Elsewhere empirical evidence from natural disasters, such as the 2004 tsunami in India and Indonesia, reported a stark gender disparity in mortality rates, with significantly more women than men dying,(3) These discrepancies are not solely attributable to differences in physical abilities but may be deeply embedded in the gendered dimensions of disaster impact and response

The disruption of health service access following Cyclone Freddy disproportionately affected women, particularly those with chronic illnesses or requiring maternal health services. Women recounted challenges accessing asthma medication and maternal care, as has been reported consistently in other studies, both in Malawi and in other similar settings(12–14). Even though access to health services was made available in the aftermath of the disaster, women reported avoiding accessing these services for fear of contracting infections in overcrowded places. This was also observed in other studies where the use of maternal and reproductive health services immediately decreased after the occurrence of Cyclone Freddy in Malawi(12) Men, in contrast, were less likely to cite personal health needs but expressed concern for the health of their spouses and children showing a dual lens where women navigate both personal and caregiving health burdens, while men experience indirect distress through familial health disruptions.

Access to clean water and safe sanitation was a widespread concern, particularly for displaced women. As previously reported by Red Cross on WASH challenges in the aftermath of the 2022 Cyclone Ana and Gombe, which destroyed a total of 337 boreholes, 8 gravity-fed water schemes, 206 water taps and 53,962 latrines, which resulted in the contamination of community wells(11,15). Whereas in the 2023 tragedy, Cyclone Freddy left 944,784 people with no access to WASH services and destroyed a total of 90,809 latrines(9) After Cyclone Freddy, many latrines in the survivor camps were mixed gender and lacked privacy, causing discouraging use mostly by women. In our study, men’s narratives often focused on infrastructure failures and unclean toilets but less on privacy. This contrast also highlights how WASH challenges, while shared, are experienced differently due to gendered social expectations. Similarly, menstrual hygiene

management emerged as a significant but often overlooked concern. While some key informants acknowledged this gap and noted efforts to distribute menstrual supplies, these interventions were often delayed.

Both survivor and stakeholder accounts confirmed the occurrence of transactional sex, coercion by aid providers or men within camps after Cyclone Freddy also as reported by UNICEF.(16) . Although some men denied security failures, others admitted that policing was inadequate. These perspectives reflect both denial and acknowledgment of GBV's pervasiveness, reinforcing the need for survivor-cantered protection services. The exploitation of women and girls during disasters has been widely reported by other scholars, including Brown S et al, who have reported harassment, trafficking and sexual exploitation after the occurrence of earthquake in Nepal (5)

Another important but less studied aspect of health impacts was mental health. The array of mental health conditions potentially exacerbated by cyclones extends beyond depression to include acute stress disorder, anxiety, adjustment disorders, PTSD, and sleep disorder (8) In our study the psychological trauma following Cyclone Freddy was universal but experienced through gendered lenses. Women frequently spoke of persistent fear, insomnia, and emotional distress, especially when recalling family deaths or the loss of their homes. Men, meanwhile, focused on economic strain, the loss of businesses, and the frustration of not being able to provide. Even though most survivors highlighted requiring mental health services, there was a gap in the provision of mental health services, as most of them did not receive the required (8,17)psychosocial support due to a shortage of trained counsellors. Scarcity of trained counsellors was also

observed in previous studies which called for professionally trained counsellors and psychologists to provide mental health services to cyclone survivors as well as survivors of any other disaster (11,15). The mental health effects of such experiences, combined with structural barriers to recovery highlight the urgency of a gender-informed mental health and livelihoods response.

The gender responsiveness of humanitarian actors varied. While some efforts were made, such as providing separate sanitation facilities, recruiting female health workers, key informants admitted that gender was not always integrated into preparedness plans and part of this was attributed to lack of gender disaggregated data. The absence of gender sensitive policies in making disaster-related decisions and ideological barriers can restrict women's access to crucial public shelters for survival, a situation highlighted by the catastrophic cyclone in Bangladesh in 1991 (13). There was consensus among key stakeholders, however, that future responses must build gender sensitivity into all phases of assessment, planning, implementation, and evaluation.

Policy Implications and Recommendations

The gendered experiences of Cyclone Freddy survivors highlight critical gaps in Malawi's disaster preparedness and response frameworks. Delayed emergency interventions were consistently cited as a barrier to life-saving care, especially for women experiencing childbirth complications or injuries. Developing rapid response protocols and improving early warning systems, especially for at-risk areas, should be a national priority.

Relief distribution processes also lacked gender sensitivity. Women struggled to access essential items such as sanitary pads, contraceptives, and private WASH facilities. Future aid efforts must include gender-responsive needs assessments, with dedicated provisions for women, adolescent girls, and female-headed households. Aid actors should receive training on equitable targeting and ensure community involvement in distribution planning to minimize exclusion.

Long-term recovery efforts should prioritize livelihoods and mental health as these have indirect implications on physical health. Both women and men reported losing businesses, land, or income sources. Gender-inclusive livelihood programs that offer capital, agricultural inputs, and vocational support are needed. Equally, mental health services must be scaled up, especially those that address trauma and gender-based violence. Community-based psychosocial support models can offer accessible pathways for recovery.

Finally, institutional coordination must improve. Fragmented roles among local agencies and aid partners weakened the response. Strengthening disaster management structures with clear gender mandates can ensure coherent, inclusive responses. More importantly, women should be actively involved in disaster risk governance at all levels, from preparedness to policymaking to ensure resilience in future crises.

Conclusion

The findings of this study demonstrate that Cyclone Freddy’s impact in Malawi was neither gender-neutral nor uniformly experienced. Importantly, gaps in humanitarian

preparedness and response often amplified these disparities. Beyond short-term aid, long-term strategies must ensure that they are informed by gender specific needs assessment. Future responses must move beyond generalized support to address the differentiated impacts of crises on men and women, with active participation of affected communities in shaping recovery plans.

Acknowledgements

We thank all the survivors of Cyclone Freddy who participated in this study and shared their experiences. We are also grateful to the local authorities, camp coordinators, and humanitarian actors who facilitated access and supported the fieldwork. Special thanks go to the key informants from government and non-governmental organizations for their valuable insights. We acknowledge the field research team for their dedication and professionalism throughout data collection

Reference

1. OPERATION UPDATE 1 Malawi | Tropical Cyclone Freddy.
2. O'Brien G, O'Keefe P, Rose J, Wisner B. Climate change and disaster management. Disasters. 2006 Mar;30(1):64–80.
3. Chitongo L, Tagarirofa J, Chazovachii B, Marango T. Gendered impacts of Climate Change in Africa: The case of Cyclone Idai [Internet]. Available from: https://www.researchgate.net/publication/343386077
4. Department of Disaster Management Affairs MALAWI: Post Tropical Storm ANA Response report.
5. Brown S, Budimir M, Upadhyay Crawford S, Clements R, Sneddon A. GENDER AND AGE INEQUALITY OF DISASTER RISK. Gender and Age Inequality; 2019.
6. Pearse R. Gender and climate change. Wiley Interdiscip Rev Clim Change. 2017 Mar 1;8(2).
7. Gender, climate change and health. World Health Organization; 2014. 36 p.
8. Makwana N. Disaster and its impact on mental health: A narrative review. J Family Med Prim Care. 2019;8(10):3090.
9. Braka F, Daniel EO, Okeibunor J, Rusibamayila NK, Conteh IN, Ramadan OPC, et al. Effects of tropical cyclone Freddy on the social determinants of health: the narrative review of the experience in Malawi. BMJ Public Health. 2024 Apr;2(1):e000512.
10. Braun V, Clarke V. Using thematic analysis in psychology.
11. Kangoma M, Lubanga FA, Bwanali AN. Mental health impact of the 2023 Tropical Cyclone Freddy and Southern region mudslides on Malawian women: the neglected humanitarian crisis. International Journal of Surgery: Global Health. 2023 Sep;6(5).

12. Twabi HH, Jafali J, Mndala L, Riches J, Monk EJM, Phiri D, et al. Cyclone Freddy and its impact on maternal health service utilisation: Cross-sectional analysis of data from a national maternal surveillance platform in Malawi. PLOS Global Public Health. 2024 Aug 28;4(8).
13. Rezwana N. Disasters and access to healthcare in the coastal region of Bangladesh: a gendered analysis.
14. Ayeb-Karlsson S. When the disaster strikes: Gendered (im)mobility in Bangladesh. Clim Risk Manag. 2020 Jan 1;29.
15. Chitongo L, Tagarirofa J, Chazovachii B, Marango T. Gendered impacts of Climate Change in Africa: The case of Cyclone Idai. Vol. 3, The Fountain-Journal of Interdisciplinary Studies.
16. van Daalen K, Jung L, Dhatt R, Phelan AL. Climate change and gender-based health disparities. Vol. 4, The Lancet Planetary Health. Elsevier B.V.; 2020. p. e44–5.
17. Mucherera B, Spiegel S. Forced displacement: critical lessons in the protracted aftermath of a flood disaster. GeoJournal. 2022 Oct 1;87(5):3855–75.
