# Supplementary material for: Living through Cyclone Freddy: “Gendered Health Impacts and Coping Responses in Malawi”
Source: PLOS Glob Public Health. 2026 May 7;6(5):e0005799. doi: 10.1371/journal.pgph.0005799 (PMC13152185; doi:10.1371/journal.pgph.0005799)
Supplement: Attachment — Submitted filename: Reviewer_Comments_MAH.docx [file pgph.0005799.s001.docx]

| Methodology section:  Need to clearly detailing who the interviewers were (e.g., "Independent academic researchers with no affiliation to the aid distribution teams").  Mention if you used private tents, partitioned areas, or off-site locations to ensure the survivors felt safe speaking about Gender-Based Violence (GBV) without being overheard by camp officials or male relatives.  Clarify if you provided a "distress protocol" (e.g., "Interviews were paused if participants became emotional, and a counselor was on standby"). |  |
| --- | --- |
| Line 156 (Participant Characteristics): Incomplete Sentence  The sentence regarding the mean age in Chiradzulu ends abruptly: "The mean age (SD) of the participants was 46.2 (14.6) years in Blantyre and 35.6 (12.9) year…? |  |
| Line 161: You list six major themes in the summary (Lines 161–164), but the headers that follow do not match these exactly (e.g., Header 1 is "Immediate Physical Health and Displacement," but the summary list says "Impact of Cyclone Freddy").  Need to arrange the headers in the Results section (Lines 167, 208, 264, etc.) use the exact wording provided in the thematic summary list to maintain structural integrity |  |
| Line 208 vs. Line 228: The heading for Section 2 is "Access to Maternal Health Services," yet the initial paragraphs (Lines 209–227) focus on general health services like malaria, BP, and HIV not those specific maternal health services.  May be rename the section or move the general health findings to a separate subsection so the "Maternal" heading remains accurate. |  |
| Lines 240–245 : A participant mentions a wife miscarrying "after the accident…?."  it is important to clarify if the "accident" refers to a specific physical trauma during the cyclone or the cyclone event itself. |  |
| Line 252 : The paragraph starting at Line 252 ("Reproductive health needs were severely disrupted...") repeats many of the points already made in the preceding section.  Merge these observations. Redundancy in qualitative results can give the impression of "filler" text rather than distinct findings. |  |
| Line 271–277 : The quote from the Male survivor (Line 271) discusses low water pressure from the "water board" and geographic conditions. It is unclear if he is describing the pre-disaster permanent infrastructure or the temporary camp conditions.  Specify the context of the quote. If survivors are critiquing the camp, the mention of "water boards" and "piped water" needs context regarding whether the camp was an official site or an informal settlement. |  |
| Line 281–283 : The text states "toilets for both males and females" (implying separation) but then notes women used "male toilets."  Clarify if the toilets were intended to be separate but weren't labeled, or if the sheer volume of people made the separation impossible to maintain. This is a key distinction for "gender-responsive" analysis. |  |
| Line 322–326: The text notes women were "not sex workers" but engaged in "survival strategies."  Use consistent terminology. Usually, in humanitarian contexts, this is defined as Sexual Exploitation and Abuse (SEA) or Transactional Sex. Clearly state that these acts were a result of systemic aid failure rather than personal choice to strengthen your argument on vulnerability. |  |
| Line 342–343: You mention "few incidences of rape and abuse of power were reported."  In qualitative research, "few" is subjective. Does this mean few participants mentioned it, or few official reports were made? Given the sensitivity, specify if this surfaced through IDIs or if it was documented in official KII data. |  |
| Line 379–381 : "Delayed" Support  To provide actionable feedback for policymakers, define "late." Does this mean weeks, months, or after the camps were already being decommissioned? |  |
| Lines 528–532: While the study correctly identifies the "gendered lens" of trauma (women focusing on family/fear and men on livelihoods), the discussion would be significantly strengthened by exploring why this occurs through the lens of socially constructed roles. |  |
| Lines 541–549: The discussion on humanitarian responsiveness is a bit descriptive. It notes that gender was "not always integrated" but needs to more aggressively address the structural reasons for this failure.  You mention a "lack of gender-disaggregated data" (Line 544) and "ideological barriers" (Line 545). To make this actionable, the discussion should contrast the Malawi National Resilience Strategy or existing disaster policies with the actual implementation described by your Key Informants. |  |
