# Supplementary material for: Living through Cyclone Freddy: “Gendered Health Impacts and Coping Responses in Malawi”
Source: PLOS Glob Public Health. 2026 May 7;6(5):e0005799. doi: 10.1371/journal.pgph.0005799 (PMC13152185; doi:10.1371/journal.pgph.0005799)
Supplement: Attachment — Submitted filename: Responses to Reviewers.docx [file pgph.0005799.s004.docx]

*10^th^ March 2026*

Dear Editorial team,

***Re: "PGPH-D-25-03980 Living Through Cyclone Freddy: “Gendered Health Impacts and Coping Responses in Malawi***”

Thank you for the opportunity to submit a revised version of our manuscript. We are deeply appreciative to the editor and reviewers for their thoughtful comments and suggestions. These specific comments enabled us to improve and strengthen the manuscript. Please find below our line-by-line response to the highlighted journal requirements and reviewer’s comments (journal requirements and reviewer comments are in italics).

Thank you for your consideration of this re-submission and please let us know if more information is required.

Yours sincerely,

Chipiliro Payesa (on behalf of the team)

Chipiliro Payesa,

MSc in Reproductive and Sexual Health Research
FemAnalytica Research Director

E: [payesac@femanalytica.org](mailto:payesac@femanalytica.org)

**SPECIFIC JOURNAL REQUIREMENTS RESPONSES**

Journal Requirements:

1. Please provide additional details regarding participant consent. In the ethics statement in the Methods and online submission information, please ensure that you have specified (1) whether consent was informed and (2) what type you obtained (for instance, written or verbal, and if verbal, how it was documented and witnessed). If your study included minors, state whether you obtained consent from parents or guardians. If the need for consent was waived by the ethics committee, please include this information.

If you are reporting a retrospective study of medical records or archived samples, please ensure that you have discussed whether all data were fully anonymized before you accessed them and/or whether the IRB or ethics committee waived the requirement for informed consent. If patients provided informed written consent to have data from their medical records used in research, please include this information.

***Response:*** ***Thank you for this clarification request. We confirm that informed written consent was obtained from all participants prior to data collection. All participants were adults aged 18 years and above, and therefore parental or guardian consent was not required. Participants were provided with detailed information about the study and informed that participation was voluntary and that they could withdraw at any time without consequence. We have revised the Ethics Statement in the Methods section to explicitly clarify that written informed consent was obtained from all participants.***

2. Please amend your detailed Financial Disclosure statement. This is published with the article. It must therefore be completed in full sentences and contain the exact wording you wish to be published.

i. Please clarify all sources of financial support for your study. List the grants, grant numbers, and organizations that funded your study, including funding received from your institution. Please note that suppliers of material support, including research materials, should be recognized in the Acknowledgements section rather than in the Financial Disclosure. 
ii. State the initials, alongside each funding source, of each author to receive each grant. For example: "This work was supported by the National Institutes of Health (####### to AM; ###### to CJ) and the National Science Foundation (###### to AM)."
iii. State what role the funders took in the study. If the funders had no role in your study, please state: “The funders had no role in study design, data collection and analysis, decision to publish, or preparation of the manuscript.”
iv. If any authors received a salary from any of your funders, please state which authors and which funders.

***Response:*** ***Thank you. Please see detailed financial disclosure statement below:***

***This work was supported by a research subgrant awarded to FemAnalytica through Simon Fraser University as part of the Gender and Public Health Emergencies Small Grant Program, funded by the Bill & Melinda Gates Foundation.***  ***The funders had no role in the study design, data collection and analysis, decision to publish, or manuscript preparation.***

3. Please ensure that your Ethics Statement is available in its entirety at the beginning of your Methods section, under a subheading 'Ethics Statement'.

***Response: Thank you for this clarification. We have revised the manuscript to ensure that the full Ethics Statement appears at the beginning of the Methods section under the subheading “Ethics Statement.”***

4. In the online submission form, you indicated that “Data is available upon request”.

All PLOS journals now require all data underlying the findings described in their manuscript to be freely available to other researchers, either 
1. In a public repository, 
2. Within the manuscript itself, or 
3. Uploaded as supplementary information.

This policy applies to all data except where public deposition would breach compliance with the protocol approved by your research ethics board. If your data cannot be made publicly available for ethical or legal reasons (e.g., public availability would compromise patient privacy), please explain your reasons by return email and your exemption request will be escalated to the editor for approval. Your exemption request will be handled independently and will not hold up the peer review process, but will need to be resolved should your manuscript be accepted for publication. One of the Editorial team will then be in touch if there are any issues.

If the reviewer comments include a recommendation to cite specific previously published works, please review and evaluate these publications to determine whether they are relevant and should be cited. There is no requirement to cite these works unless the editor has indicated otherwise.

***Response: Thank you for this request. Due to the sensitive nature of the qualitative data collected, which includes personal experiences of displacement, gender-based violence, and health challenges following Cyclone Freddy, the interview transcripts cannot be made publicly available to protect participant confidentiality and privacy. This restriction is consistent with the ethical approval granted by the Malawi National Commission for Science and Technology. However, de-identified excerpts of the data are presented in the manuscript to support the study findings. At this stage, we will pursue the exemption request by email to the editor.***

**SPECIFIC REVIEW RESPONSES**

**REVIEWER #1**

General comments*:* This is a very interesting study, valuable new information and has the potential to meet the standards. There are just a few minor but important issues to address*.*

***Response: We thank the reviewer for the thoughtful and constructive feedback and for recognizing the importance of this study. We appreciate the suggestions to strengthen the conceptual framing of gender and deepen the analysis of differentiated experiences during Cyclone Freddy. We have carefully revised the manuscript to address these comments. Our responses are provided below***.

Reviewer comment:

The reviewer notes that in some sections the manuscript treats gender as synonymous with women and girls and suggests strengthening the gender analysis by examining how both women and men experience disasters differently.

***Response:***

***We agree with the reviewer that gender should not be treated solely as synonymous with women and girls. We have revised the manuscript throughout to clarify that gender is understood as a social determinant shaping the experiences of both women and men during disasters. In particular, we have strengthened language in the Abstract, Introduction, and Discussion sections to highlight differentiated gendered experiences rather than focusing exclusively on women and girls.***

***In the Results section, we now more explicitly contrast how women often discussed caregiving responsibilities and disruptions to health services, while men more frequently described livelihood loss and economic pressures. These revisions help ensure that gender is treated as a relational concept rather than a proxy for women alone.***

Reviewer comment:

The reviewer suggests exploring more nuanced gender differences, for example how men discuss care and how women discuss livelihoods, or how gender may influence access to services during crises.

***Response:***

***We appreciate this suggestion and have expanded the analytical discussion to more explicitly examine how gender norms shape the ways individuals experience and articulate disaster impacts. In the Discussion section, we added text highlighting that men frequently framed their experiences through loss of livelihoods and provider roles, while women more often emphasized caregiving burdens and access to health services. We also added analysis noting the relative absence of men’s discussions of personal healthcare needs, which may reflect gendered norms around care-seeking and help-seeking behaviors***.

***Additionally, we expanded the Discussion to consider how disruptions to maternal and reproductive health services may disproportionately affect women who rely on these services as key entry points into the healthcare system during crises.***

Reviewer comment:

The reviewer suggests that the manuscript should more clearly describe baseline gender inequalities (e.g., GBV risks or structural vulnerabilities) and demonstrate how disasters exacerbate these inequalities.

***Response:***

***We agree that providing context on pre-existing gender inequalities strengthens the interpretation of the findings. We have therefore revised the Introduction to include additional discussion of baseline structural inequalities in Malawi, including gender-based violence risks, unequal access to resources, and gendered caregiving roles. These revisions help situate the study findings within the broader context of gendered vulnerabilities that existed prior to Cyclone Freddy.***

Reviewer comment:

The reviewer asks how men discuss care and whether women discuss livelihoods, suggesting that highlighting such patterns (including absences) would strengthen the analysis.

***Response:***

***To address this comment, we revised both the Results and Discussion sections to more clearly describe these patterns. The manuscript now explicitly notes that men predominantly framed disaster impacts in terms of livelihood loss and economic instability, while women more frequently discussed caregiving responsibilities, health service disruptions, and family wellbeing. We also added analytical text highlighting that the relative absence of men’s narratives around personal healthcare and women’s limited emphasis on independent livelihoods reflects gendered social expectations and role norms.***

Reviewer comment:

The reviewer notes that disasters are often described as gender-neutral events but that their impacts are gender-differentiated.

***Response:***

***We appreciate this important clarification and have revised the Introduction to avoid framing disasters as gender-neutral. The text now reflects current scholarship indicating that while disasters may affect entire populations, their impacts are shaped by gendered social structures and inequalities. We also incorporated the recommended reference to strengthen the conceptual framing of gendered disaster impacts***.

Reviewer comment:

The reviewer suggests more clearly articulating how the study advances existing knowledge.

***Response:***

***We have revised the Discussion to more clearly articulate the study’s contribution to the literature. Specifically, we emphasize that while prior research has recognized gender-differentiated disaster impacts, there remains a gap in understanding how these dynamics unfold within specific local contexts such as Malawi. By integrating survivor narratives and humanitarian stakeholder perspectives, this study provides context-specific insights into how gender norms shape both vulnerability and recovery during climate-related disasters.***

***General comment***

Thank you for the opportunity to review your manuscript, “Living Through Cyclone Freddy: Gendered Health Impacts and Coping Responses in Malawi.” The study is timely, policy-relevant

***Response***

***We thank the reviewer for the thoughtful and constructive feedback and for recognizing the importance and policy relevance of this study. We appreciate the detailed suggestions to strengthen the conceptual framing, methodological transparency, and analytical depth of the manuscript. We have carefully revised the manuscript to address these comments. Our responses to each point are provided below.***

Reviewer comment:

Gender is largely treated as a descriptive binary category rather than as a social and structural determinant shaping vulnerability, access to services, and recovery.

***Response:***

***We appreciate this important observation and have strengthened the conceptual framing of gender throughout the manuscript. In the Introduction, we now clarify that gender is treated as a social determinant of health shaped by social norms, power relations, and structural inequalities that influence vulnerability, access to services, and recovery during disasters. We also added text explaining how this conceptual perspective informed the study design, data collection, and interpretation of findings. These revisions help ensure that gender is treated as a relational and structural construct rather than solely as a binary descriptive category.***

Reviewer comment:

Participant recruitment, inclusion criteria, gender balance, data saturation, interviewer characteristics, and reflexivity need further explanation.

***Response:***

***We have expanded the Methods section to provide additional methodological detail. Specifically, we clarified the participant recruitment process within displacement camps, including collaboration with camp coordinators and the purposive sampling strategy used to identify information-rich participants. We also added inclusion criteria and described the approach used to ensure representation across gender groups. Furthermore, we clarified how thematic saturation informed the data collection process. Additional details regarding interviewer characteristics, reflexivity, and steps taken to minimize power imbalances during interviews have been included in both the Data Collection and Ethics sections.***

Reviewer comment:

More detail is needed regarding code development, team-based coding, and strategies to enhance trustworthiness.

***Response:***

***We have revised the Data Analysis section to provide a clearer description of the analytical process. The manuscript now specifies that the analysis followed Braun and Clarke’s reflexive thematic analysis approach. We describe how the initial coding framework was developed, how the codebook was refined through iterative team discussions, and how codes were systematically applied to the dataset. We also added information about strategies used to enhance the credibility and trustworthiness of the analysis, including team-based coding discussions and triangulation between survivor interviews and key informant interviews.***

Reviewer comment:

The Results section should be streamlined, quotations used more selectively, and analytical contrasts between men and women strengthened.

***Response:***

***We have revised the Results section to improve clarity and synthesis. Repetitive text has been reduced and quotations have been used more selectively to illustrate key themes. We also strengthened analytical contrasts between men’s and women’s experiences, highlighting how men frequently discussed livelihood loss and provider roles, while women emphasized caregiving responsibilities and disruptions to health services. These revisions enhance the interpretive depth of the findings.***

Reviewer comment:

The Discussion should more clearly explain how the study advances knowledge on Cyclone Freddy and gendered disaster health impacts.

***Response:***

***We have strengthened the Discussion section to better highlight the contribution of this study to existing scholarship. The revised text emphasizes how the findings extend current knowledge by providing context-specific insights into how gender norms shape vulnerability and recovery pathways in disaster settings in Malawi. We also expanded engagement with relevant literature to situate the findings within broader research on gender, climate-related disasters, and health systems.***

Reviewer comment:

More explicit discussion of participant protection, referral pathways, and limitations is needed.

***Response:***

***We have revised the Ethics section to include additional information regarding safeguards for participants discussing sensitive issues such as gender-based violence. The manuscript now describes procedures used to ensure confidentiality, minimize distress during interviews, and provide information on available support services if participants required assistance. In addition, we have added a dedicated Limitations section that reflects on methodological constraints and ethical considerations associated with conducting research in disaster-affected populations.***

***Reviewer 3***

# *General Response to Reviewer*

***We thank the reviewer for the careful reading of the manuscript and for the detailed suggestions to improve methodological transparency, structural clarity, and analytical precision. We have revised the manuscript accordingly and respond to each comment below.***

| Reviewer Comment: Methodology section:  Need to clearly detailing who the interviewers were (e.g., "Independent academic researchers with no affiliation to the aid distribution teams").  Mention if you used private tents, partitioned areas, or off-site locations to ensure the survivors felt safe speaking about Gender-Based Violence (GBV) without being overheard by camp officials or male relatives.  Clarify if you provided a "distress protocol" (e.g., "Interviews were paused if participants became emotional, and a counselor was on standby").  ***Response:***  ***We appreciate this important suggestion and have revised the Data Collection and Ethics sections to provide additional detail. Specifically, we now clarify that interviews were conducted by trained qualitative researchers with experience in gender and public health research and no affiliation with aid distribution teams. We also added information describing how interviews were conducted in private or semi-private locations within or near the camps to ensure participants could speak freely without being overheard. In addition, we included a description of the distress protocol used during interviews, noting that interviews were paused if participants became distressed and that participants were provided with information on available support services when needed*** |  |
| --- | --- |
| Reviewer comment Line 156 (Participant Characteristics): Incomplete Sentence  The sentence regarding the mean age in Chiradzulu ends abruptly: "The mean age (SD) of the participants was 46.2 (14.6) years in Blantyre and 35.6 (12.9) year…?  ***Response:***  ***Thank you for noting this oversight. The sentence has been corrected to fully report the mean age and standard deviation for participants in both districts.*** |  |
| Reviewer comment Line 161:  You list six major themes in the summary (Lines 161–164), but the headers that follow do not match these exactly (e.g., Header 1 is "Immediate Physical Health and Displacement," but the summary list says "Impact of Cyclone Freddy").  Need to arrange the  headers in the Results section (Lines 167, 208, 264, etc.) use the exact wording provided in the thematic summary list to maintain structural integrity  **Response:**  **We have revised the Results section headers so that they now match the thematic summary list exactly. This ensures structural consistency between the thematic overview and the detailed findings.** |  |
| Reviewer comment  Line 208 vs. Line 228: The heading for Section 2 is "Access to Maternal Health Services," yet the initial paragraphs (Lines 209–227) focus on general health services like malaria, BP, and HIV not those specific maternal health services.  May be rename the section or move the general health findings to a separate subsection so the "Maternal" heading remains accurate.  ***Response:***  ***We have revised this section to improve clarity. The context has been adjusted to better reflect the heading of acess to maternal and reproductive health findings.*** |  |
| Reviewer comment  Lines 240–245 : A participant mentions a wife miscarrying "after the accident…?."   it is important to clarify if the "accident" refers to a specific physical trauma during the cyclone or the cyclone event itself. |  |
| ***Response:***  ***We have revised the text to clarify that the participant was referring to the cyclone event and related displacement conditions, rather than a separate accident.***  Reviewer comment |  |
| Line 252 : The paragraph starting at Line 252 ("Reproductive health needs were severely disrupted...") repeats many of the points already made in the preceding section.  Merge these observations. Redundancy in qualitative results can give the impression of "filler" text rather than distinct findings.  ***Response:***  ***We have revised the section to remove redundancy and merge overlapping observations to improve clarity and conciseness.***  Reviewer comment |  |
| Line 271–277 : The quote from the Male survivor (Line 271) discusses low water pressure from the "water board" and geographic conditions. It is unclear if he is describing the pre-disaster permanent infrastructure or the temporary camp conditions.  Specify the context of the quote. If survivors are critiquing the camp, the mention of "water boards" and "piped water" needs context regarding whether the camp was an official site or an informal settlement.  ***Response:***  ***We have clarified placed a more relevant quote that expressesto water access conditions experienced during displacement.*** |  |
| Reviewer comment  Line 281–283 : The text states "toilets for both males and females" (implying separation) but then notes women used "male toilets."  Clarify if the toilets were intended to be separate but weren't labeled, or if the sheer volume of people made the separation impossible to maintain. This is a key distinction for "gender-responsive" analysis.  ***Response:***  ***We have revised the text to clarify that although sanitation facilities were intended to be gender-separated, the high population density in the camps and limited infrastructure made it difficult to maintain separation in practice.*** |  |
| Reviewer comment  Line 322–326: The text notes women were "not sex workers" but engaged in "survival strategies."  Use consistent terminology. Usually, in humanitarian contexts, this is defined as Sexual Exploitation and Abuse (SEA) or Transactional Sex. Clearly state that these acts were a result of systemic aid failure rather than personal choice to strengthen your argument on vulnerability.  ***Response:***  ***We have revised the text to use more consistent terminology and clarify that these experiences reflect transactional sex and sexual exploitation arising from structural vulnerabilities during displacement, rather than individual choice***.  Reviewer comment |  |
| Line 342–343: You mention "few incidences of rape and abuse of power were reported."  In qualitative research, "few" is subjective. Does this mean few participants mentioned it, or few official reports were made? Given the sensitivity, specify if this surfaced through IDIs or if it was documented in official KII data.  ***Response:***  ***We have revised the wording to clarify that this observation refers to instances mentioned during interviews with survivors and key informants, rather than official incident reports***. |  |
| Reviewer comment  Line 379–381 : "Delayed" Support  To provide actionable feedback for policymakers, define "late." Does this mean weeks, months, or after the camps were already being decommissioned?  ***Response:***  ***We have revised the text to clarify that participants reported mental health and psychosocial support becoming available several weeks after displacement, which many felt was later than needed.*** |  |
| Reviewer comment  Lines 528–532: While the study correctly identifies the "gendered lens" of trauma (women focusing on family/fear and men on livelihoods), the discussion would be significantly strengthened by exploring why this occurs through the lens of socially constructed roles.  ***Response:***  ***We have expanded this section of the Discussion to interpret these differences through the lens of gender norms and socially constructed roles. The revised text now explains how caregiving responsibilities and provider expectations shape the ways women and men experience and articulate disaster-related distress.*** |  |
| Reviewer comment  Lines 541–549: The discussion on humanitarian responsiveness is a bit descriptive. It notes that gender was "not always integrated" but needs to more aggressively address the structural reasons for this failure.  You mention a "lack of gender-disaggregated data" (Line 544) and "ideological barriers" (Line 545). To make this actionable, the discussion should contrast the Malawi National Resilience Strategy or existing disaster policies with the actual implementation described by your Key Informants. |  |

***Response:***

***We have revised the Discussion to strengthen the analysis of structural barriers to gender-responsive disaster response. The revised section now discusses gaps between existing national disaster policies and their implementation during Cyclone Freddy, highlighting challenges such as limited gender-disaggregated data, institutional capacity constraints, and coordination gaps identified by key informants***.

Reviewer 4

***We thank the reviewer for the positive assessment of the manuscript and for recognizing its contribution to understanding gendered health impacts of disasters in low-resource settings. We appreciate the constructive suggestions to improve the clarity and structure of the manuscript. Our responses to each point are provided below.***

Reviewer comment:

The findings could benefit from clearer structure by rephrasing and organizing the headings and subheadings.

***Response:***

***Thank you for this helpful suggestion. We have revised the Results section to improve structural clarity by refining the wording of the main themes and aligning the section headings with the thematic summary presented earlier in the section.***

Reviewer comment:

The discussion would benefit from breaking into shorter paragraphs to improve readability.

**Response:**

**We have revised the Discussion section to improve readability by breaking longer paragraphs into shorter ones and improving the logical flow between ideas.**

Reviewer comment:

Some sentences should be simplified for clarity.

***Response:***

***We carefully reviewed the manuscript and revised several long sentences throughout the text to improve clarity and readability.***

Reviewer comment:

The manuscript should mention limitations, including the challenges of conducting research in disaster settings and potential biases.

***Response:***

***We agree with this suggestion and have added a dedicated Limitations section to the manuscript. This section discusses methodological considerations including recall bias, challenges associated with conducting research in disaster settings, and the potential influence of social desirability bias when discussing sensitive topics such as gender-based violence.***

***Reviewer 5***

***General response: We thank the reviewer for the thoughtful and constructive feedback and for recognizing the contribution of this study to the literature on gender-responsive humanitarian assistance in disaster settings. We appreciate the detailed suggestions provided to improve methodological clarity, organization of results, and overall readability. We have revised the manuscript accordingly and respond to each point below.***

Reviewer comment

Reviewer #5: This qualitative study describes differences in men's and women's experiences during a natural disaster and resulting displacement, as well as experiences with humanitarian assistance in Malawi. The findings contribute the global health literature on the importance of gender-responsive humanitarian assistance and also documenting the unique burdens that populations may experience based on their gender/sex, age, disability status, and so forth.

Overall, the manuscript would benefit from careful copy-editing to address errors in punctuation and formatting.

***Response:***

***Thank you for this suggestion. We have carefully reviewed the manuscript and conducted thorough copy-editing to improve punctuation, formatting consistency, and overall clarity throughout the text.***

Reviewer comment

In the presentation of the Results, some of the thematic statements were out of sequence with the quotes being presented. I recommend that the authors take care to ensure that the organization of summarizing the themes and then presenting the illustrative quotes has a clear and logical flow throughout.

***Response:***

***We have revised the Results section to improve the logical flow between thematic summaries and illustrative quotes. The presentation of themes and supporting quotations has been reorganized to ensure that each quote directly follows and supports the thematic statement being described***.

Reviewer comment

The weakest aspect of the manuscript was the methods section, and I offer several suggestions to improve this section below:

Study design and setting:
- insert "cross-sectional" before "qualitative study" in the opening sentence (Line 91)
- Line 95 in this section, change "comprehensive view" to "comparative view"
***Response:***

***We have revised the Study Design and Setting section accordingly. The opening sentence now describes the study as a cross-sectional qualitative study, and the phrase “comprehensive view” has been replaced with “comparative view” to more accurately describe the comparison between urban and rural study sites.***

Reviewer comment

Sampling
- in the first sentence, offer a methods reference for purposive sampling procedure

***Response:***

***We have added reference in this section to support purposive sampling. We have also expanded the explanation of how thematic saturation informed the data collection process, including relevant methodological references to support this approach.***

Reviewer comment

- at Line 105 in this section, add "quota" or "stratified" before the word "sampling" in that sentence

***Response:***

***We have clarified that a stratified purposive sampling approach was used to ensure representation across gender and demographic groups***

Reviewer comment

- The use of "saturation" as a tool to determine when to stop recruitment and stop interviewing participants is not always warranted; In many cases, there are logistical constraints such as funding, research assistance, timeline, access to participants, and so forth. In others, there are methodological considerations that guide the decision regarding sampling for saturation, but there are diverse approaches. The authors should discuss - if they are going to describe saturation for sampling - what approach they are using or modeling their approach after, using appropriate references to support their methodological decision-making.

***Response***

***Thank you for this helpful comment. Our intention was not to suggest that participant recruitment was determined solely by saturation. Rather, the sample size was guided by the study objectives, field access, and the scope of the study. We have revised the Sampling section to clarify this point and added methodological references to support the purposive sampling approach used in the study. We also clarified that during analysis, interviews were observed to yield recurring themes, indicating that the data provided sufficient coverage of the study topics.***

Reviewer comment

Data Collection
- the authors reference "designated survivor camps" (Line 118); it would be helpful to the readers to understand more about the settings and also the approvals/permissions that were granted to gain entree into the camps and access to the residents as there are conventions regarding research within these displaced populations (e.g., SPHERE standards, etc.)

***Response:***

***We have expanded the Data Collection section to describe the study setting more clearly and explain how access to the displacement camps was obtained through coordination with local authorities and camp management. We have also included references to established ethical guidance for conducting research in humanitarian settings to demonstrate alignment with recommended practices.***

Reviewer comment

- there are also guidance for conducting research with populations affected by humanitarian crises that are used in global health, but none are referenced in the methods section. The authors should add this information and indicate whether or not they followed these recommendations (e.g., ERLHA resources).

**Response:**

**Thank you for this important suggestion. We have revised the Data Collection and Ethics sections to acknowledge established guidance for conducting research in humanitarian and disaster settings. Specifically, we now reference ethical guidance commonly used in global health research with crisis-affected populations and clarify that the study procedures were designed to align with these principles, including ensuring voluntary participation, protecting confidentiality, and minimizing potential harm to participants**.

Reviewer comment

- The authors are treating "semi-structured interviews" (a type of interview) with "key informant interviews" (a type of participant) as in the same category in Line 119-119. Key informants can be interviewed using semi-structured interviews, so the sentence as written does not make sense. Please revise.
***Response:***

***We appreciate this clarification. We have revised the sentence in the Data Collection section to clearly state that semi-structured interviews were conducted with both cyclone survivors and key informants, rather than presenting them as separate interview types. This revision clarifies the methodological approach and ensures consistency with qualitative research terminology.***

Reviewer comment

Ethics Statement
- Here is a good place to discuss using recommended procedures for working with populations affected by disasters and displacement

***Response:***

***We have revised the Ethics Statement to include additional information on ethical considerations when conducting research with disaster-affected populations, including procedures used to ensure voluntary participation, confidentiality, and participant protection during discussions of sensitive topics.***

Reviewer comment

Data Analysis
- More description of the "experienced researchers" is needed here, perhaps in the form of a positionality statement and indicating who among the author team is being included in this group. A statement of positionality or researcher reflexivity should be moved to the Ethics paragraph.

***Response***

***Thank you for this helpful suggestion. We have revised the manuscript to provide additional detail regarding the researchers involved in the data analysis, clarifying their experience in qualitative research and public health. We also added a brief positionality statement describing the research team’s professional backgrounds and familiarity with the local context.***

Reviewer comment

- "thematic analysis" is too generic to describe the process of analysis, and there should be methodological references throughout the Methods sub-sections to indicate that the authors are following accepted methodological procedures form the peer-reviewed literature. Please add more details.

***Response***

***Thank you for this helpful comment. We have revised the Data Analysis section to provide a more detailed description of the analytical procedures used in the study. Specifically, we now clarify that the analysis followed the reflexive thematic analysis approach described by Braun and Clarke and added appropriate methodological references. We also expanded the description of the analytical process to explain how transcripts were reviewed, codes were developed and refined, and how themes were constructed through iterative discussions among the research team. These revisions help clarify the methodological rigor of the analysis.***

Reviewer comment

- Add "applied the code book to the data" in Line 147 in that first sentence (The research team ...... then created....)

***Response:***

***Thank you for this helpful suggestion. We have revised the sentence in the Data Analysis section to clarify that after developing the codebook, the research team systematically applied it to the dataset before generating themes and sub-themes. This revision improves clarity regarding the sequence of the analytical proces***

Reviewer comment

- Qualitative analysis is not conducted to ensure the "validity of the findings"; what makes more sense here (Line 148) would be a more detailed description of the inter-rater reliability process (whether that was done quantiatively or qualitatively) and the procedures in a way that it could be replicated based on what is provided in the Methods section.

***Response:***

***Thank you for this helpful comment. We agree that the term “validity” may not be appropriate in this context. We have revised the Data Analysis section to replace this wording and instead describe the procedures used to strengthen the credibility of the analysis. Specifically, we clarified that multiple members of the research team reviewed the transcripts, applied the codebook to the data, and discussed coding decisions to ensure consistency in interpretation. We also describe how discrepancies in coding were discussed and resolved through team consensus. These revisions provide greater transparency regarding the analytical procedures used in the study.***

Reviewer comment

Line 161 - Revise this sentence, which is confusing. The authors created the themes, themes do not "emerge" on their own.
***Response:***

**We agree and have revised the sentence to clarify that themes were developed through iterative analysis and interpretation of the data**

Reviewer comment

Results

Line 169 - instead of "disparities", what is being described here is actually "differences" for men and women
Line 170 - more context is needed to understand the circumstances of separation during displacement
Line 183 - change prioritized to "spoke about" in this sentence
Lines 196-7 - This sentence would be better placed in the Discussion

Again.... there are many places where the summative statements in the Results and the quotes don't flow well or seem out of order; revise.

***Response:***

***We have revised the Results section to address these points. Specifically, we replaced “disparities” with “differences,” clarified the circumstances surrounding family separation during displacement, replaced interpretive language such as “prioritized” with more descriptive phrasing, and moved analytical interpretations to the Discussion section where appropriate.***
