# Supplementary material for: Living through Cyclone Freddy: “Gendered Health Impacts and Coping Responses in Malawi”
Source: PLOS Glob Public Health. 2026 May 7;6(5):e0005799. doi: 10.1371/journal.pgph.0005799 (PMC13152185; doi:10.1371/journal.pgph.0005799)
Supplement: Attachment — Submitted filename: Responses to Reviewer.docx [file pgph.0005799.s005.docx]

*16^th^ March 2026*

Dear Editorial team,

***Re: "PGPH-D-25-03980 Living Through Cyclone Freddy: “Gendered Health Impacts and Coping Responses in Malawi***”

Thank you for the opportunity to submit a revised version of our manuscript. We are grateful to the editor and reviewers for their careful assessment and constructive feedback.

In this revision, we have addressed the remaining comment from Reviewer #1 by further clarifying the conceptualization of gender as a relational and context-specific construct, and by adding brief text in the Introduction and Discussion to better situate our findings within the Malawian context.

All other reviewers indicated that their comments had been adequately addressed. A detailed response to the remaining comment is provided below.

Thank you for your consideration of our revised manuscript.

Yours sincerely,

Chipiliro Payesa (on behalf of the team)

Chipiliro Payesa,

MSc in Reproductive and Sexual Health Research
FemAnalytica Research Director

E: [payesac@femanalytica.org](mailto:payesac@femanalytica.org)

**SPECIFIC JOURNAL REQUIREMENTS RESPONSES**

Journal Requirements:

1. If the reviewer comments include a recommendation to cite specific previously published works, please review and evaluate these publications to determine whether they are relevant and should be cited. There is no requirement to cite these works unless the editor has indicated otherwise.

***Response***

***The reviewer did not recommend specific additional citations; however, we have incorporated relevant contextual clarification within the manuscript where appropriate*.**

2. Please review your reference list to ensure that it is complete and correct. If you have cited papers that have been retracted, please include the rationale for doing so in the manuscript text, or remove these references and replace them with relevant current references. Any changes to the reference list should be mentioned in the rebuttal letter that accompanies your revised manuscript. If you need to cite a retracted article, indicate the article’s retracted status in the References list and also include a citation and full reference for the retraction notice.

***Response***

***We have reviewed the reference list to ensure completeness and accuracy. No retracted articles were identified, and no further changes were required***.

**SPECIFIC REVIEW RESPONSES**

**REVIEWER #1**

**very valuable study, findings make sense but just to urge care in terms of gender that gender is not synonymous with women and girls. If accepted that gender norms and roles are all genders and interactions, expectations and consequences are embedded in a local context (which varies across all context).
Adding a sentence or two in the framing and discussion or conclusion to emphasize this point about gendered embedded in the local context would bring this out more.**

***Response:***

***We thank the reviewer for this thoughtful and constructive comment. We agree that gender should not be treated as synonymous with women and girls, and that gendered experiences are embedded within locally specific social norms, roles, and expectations.***

***Building on revisions made in the previous round, we have further refined the manuscript to more explicitly reflect gender as a relational and context-specific construct. Specifically, we have added clarifying text in the Introduction and Discussion to emphasize that gender norms in Malawi shape the experiences of both women and men during crises.***

***In response to the reviewer’s suggestion, we have also strengthened the Discussion to situate our findings within the local gender context, noting how socially embedded expectationssuch as men’s roles as economic providers and women’s roles in caregiving may influence how individuals experience and articulate the impacts of disasters.***

***We further highlight the importance of contextually grounded and locally informed humanitarian responses, including greater engagement with Malawian stakeholders, to ensure that disaster preparedness and response efforts are responsive to these gendered dynamics.***
